# Hemophagocytic Lymphohistiocytosis Masquerading as Alcoholic Hepatitis

**DOI:** 10.14309/crj.0000000000000495

**Published:** 2020-12-10

**Authors:** Sawsan Abulaimoun, Kamelah Abushalha, Savio Reddymasu, Bryan Teruya, Nagendra Natarajan

**Affiliations:** 1 Department of Internal Medicine, Creighton University, Omaha, NE; 2 Doctors Without Borders, Amman, Jordan; 3 Department of Gastroenterology, Creighton University, Omaha, NE; 4 Department of Pathology, Creighton University, Omaha, NE; 5 Nebraska Cancer Specialists, Omaha, NE

## Abstract

Hemophagocytic lymphohistiocytosis is a syndrome characterized by excessive immune activation. Timely diagnosis can be challenging, and prompt treatment is the only hope for survival. We present an adult patient with a history of alcohol dependence, who presented with fatigue, bilateral lower extremity edema, and orange-colored urine. Clinical workup revealed abnormal liver function tests, elevated ferritin, cytopenia, and lymphadenopathy. Eventually, he was diagnosed with hemophagocytic lymphohistiocytosis. This case report encourages gastroenterologists to maintain a high index of suspicion when a patient presents with liver failure, hyperferritinemia, and cytopenia because they may be the first healthcare professionals to evaluate these patients.

## INTRODUCTION

Hemophagocytic lymphohistiocytosis (HLH) is a rare aggressive type of uncontrolled immune activation that is typically underdiagnosed. The HLH-2004 trial established the diagnostic criteria for HLH, which includes a constellation of clinical findings in the setting of elevated inflammatory marker expression.^1^ Studies suggest that liver involvement is present in 85% of adult patients with HLH and ranges from mild hepatitis to overt liver failure.^2^ It is not uncommon for HLH to be mistaken for other causes of liver failure. We report a patient for whom it was very tempting to assume a diagnosis of alcoholic hepatitis, but he was ultimately diagnosed with a different condition.

## CASE REPORT

A 35-year-old man with a history of alcohol abuse (1–2 pints of vodka, 5–6 times per week for over 5 years; he became abstinent 3 weeks before presentation) was directed to the gastroenterology clinic for evaluation of fatigue, bilateral lower extremity swelling, and orange-colored urine. The examination was significant in its findings of lower extremity edema and hepatosplenomegaly. Laboratory workup showed abnormal liver function-aspartate aminotransferase 295 U/L, alkaline phosphatase 505 U/L, alanine aminotransferase 230 U/L, and bilirubin 3.4 mg/dL. Further testing revealed elevated ferritin 12,799 ng/mL and iron saturation of 68%. The patient had a history of diffuse lymphadenopathy (LAP), which was discovered incidentally and was stable for the past 6 years (lymph node biopsy was performed and resulted in no definitive diagnosis, at which time the patient was lost to follow up).

The patient was admitted, and further laboratory investigations on admission revealed pancytopenia. Peripheral blood smear showed normocytic anemia, thrombocytopenia, and absolute lymphopenia. The bilirubin level was increased to 15.3 mg/dL as was the transaminase level. Ferritin was increased to 20,000 ng/mL. The international normalized ratio was prolonged at 1.5, albumin was decreased to 1.4 mg/dL, and lactate dehydrogenase was elevated at 477 U/L. We performed pan cultures, a lipid panel, autoimmune profile (antinuclear antibody, mitochondrial antibody, and antismooth muscle antibody immunoglobulin [Ig] G), hepatitis panel, and a hemochromatosis mutation panel. We also assessed alpha 1 antitrypsin, an α-fetoprotein tumor marker, viral serologies, and erythrocyte sedimentation rate and performed a bone marrow biopsy. At that time, our differential diagnosis was alcoholic hepatitis/cirrhosis vs lymphoproliferative disorder.

Positron emission tomography revealed hypermetabolic splenomegaly, mediastinal and retroperitoneal LAP, and numerous bone marrow abnormalities. Endoscopic ultrasound revealed many abnormal enlarged lymph nodes that extended from the supraclavicular region to the liver. Biopsies of the liver, spleen, and lymph nodes were normal. A bone marrow biopsy showed normocellular bone marrow (60%) with mild erythroid hyperplasia. Remarkable positive findings of the workup were as follows: Cytomegalovirus (CMV) IgM and IgG, Epstein-Barr virus (EBV) IgG, and EBV nuclear antibody (compatible with chronic EBV infection). Other viral serologic testing included tests for hepatitis viruses, human immunodeficiency virus, and parvovirus titers; these viral serology tests and Histoplasma antibody and Fungitell tests were all negative. The autoimmune workup was also negative.

Both infectious disease and hematology teams were consulted, and considering the abovementioned results, our differential diagnoses shifted to acute CMV infection and possible HLH. Treatment was initiated with dexamethasone at 20 mg intravenously daily (day 4 after admission) and ganciclovir at 240 mg twice a day (started on day 5). Soluble interleukin-2 receptors level was assessed and found to be elevated at 41,967 pg/mL (normal range: 186–2,678 pg/mL), so a clinical diagnosis of HLH was made, and the patient, we initiated etoposide at 150 mg/m^2^ (on day 7 after admission). However, unfortunately, on the same day, the patient developed multiorgan failure with acute kidney injury, ischemic hepatitis, and acute lung injury. He was intubated and passed away 48 hours later.

## DISCUSSION

Alcoholic hepatitis is a clinical syndrome characterized by jaundice and liver-related complications that occurs in the setting of prolonged heavy alcohol use. This disease is part of a spectrum that is termed alcohol-related liver disease (ALD). According to the ACG guidelines, it is important to exclude other causes before a diagnosis of ALD is determined.^3^ A history of alcohol dependence, sudden onset of jaundice, and elevated liver enzymes strongly suggested a diagnosis of ALD in our patient. On the contrary, the findings of pancytopenia, diffuse LAP, and the extreme elevation in ferritin warrant further investigations. Studies suggest that the prevalence of abdominal LAP in chronic liver diseases is approximately 38%, whereas the highest percentage (50%) is seen in chronic hepatitis C patients, although the lowest (less than 10%) is seen in ALD.^4^ Pancytopenia in the setting of ALD could be explained by hypersplenism, a direct toxic effect of alcohol on the bone marrow, liver function impairment, and autoimmune-induced damage. Extreme hyperferritinemia is unusual in the context of ALD, and the differential diagnosis includes adult-onset Still's disease, catastrophic antiphospholipid syndrome, septic shock, and HLH.^5^

HLH is characterized by generalized proliferation of histiocytes with marked hemophagocytosis. The different types of HLH based on cause are familial, autoimmune, malignancy-associated, and viral-associated HLH. According to the HLH-2004 trial, our patient met 6 of 8 diagnostic criteria including fever, splenomegaly, cytopenia, hyperferritinemia, increased triglyceride level, decreased fibrinogen, and high levels of soluble CD25. Two of the criteria were not found in this patient—bone marrow biopsy was normal and natural killer-cell activity was not measured.

Primary CMV infection in an immunocompetent host rarely causes serious illness. However, CMV represents 10.5% of all infection-related causes of the HLH in immunocompromised hosts, whereas in immunocompetent adults, only few cases have been reported in the literature—5 cases received different successful treatments, 2 cases were treated with steroids and antiviral medication, 1 with intravenous Ig, and 1 with cyclosporin and granulocyte colony-stimulating factor.^6–9^ In an interesting case of a Japanese patient with recurrent HLH, CMV was described as the primary inciting factor, and a GATA binding protein 2 mutation was identified.^10^ HLH is typically underdiagnosed, and thus, gastroenterologists should always include HLH as a possibility when hepatitis, pancytopenia, and fever are observed.

## DISCLOSURES

Author contributions: S. Abulaimoun and K. Abushalha wrote the manuscript. K. Abushalha, S. Reddymasu, B. Teruya and N. Natarajan revised the manuscript for intellectual content.

Financial disclosure: None to report.

Previous presentation: This case was presented at the American College of Gastroenterology Annual Scientific Meeting; October 5-10, 2018; Philadelphia, Pennsylvania.

Informed consent was obtained for this case report.

## References

[1] Henter JI Horne A Aricó M HLH-2004: Diagnostic and therapeutic guidelines for hemophagocytic lymphohistiocytosis. Pediatr Blood Cancer 2007;48(2):124–31. 16937360 10.1002/pbc.21039

[2] Li J Wang Q Zheng W Hemophagocytic lymphohistiocytosis: Clinical analysis of 103 adult patients. Medicine 2014;93(2):100–5. 24646466 10.1097/MD.0000000000000022 PMC4616310

[3] Singal AK Bataller R Ahn J Kamath PS Shah VH ACG clinical guideline: Alcoholic liver disease. Am J Gastroenterol 2018;113(2):175–94. 29336434 10.1038/ajg.2017.469 PMC6524956

[4] del Olmo JA Esteban JM Maldonado L Clinical significance of abdominal lymphadenopathy in chronic liver disease. Ultrasound Med Biol 2002;28(3):297–301. 11978409 10.1016/s0301-5629(01)00514-2

[5] Cohen D Mansour R Amital H Segal G “Still is not enough”—What is the differential diagnosis and the workup when the ferritin level is extremely high? [in Hebrew] Harefuah 2020;159(3):166–9. 32186785

[6] Hot A Madoux MH Viard JP Coppéré B Ninet J Successful treatment of cytomegalovirus-associated hemophagocytic syndrome by intravenous immunoglobulins. Am J Hematol 2008;83(2):159–62. 17849465 10.1002/ajh.21008

[7] Bonnecaze AK Willeford WG Lichstein P Ohar J Acute cytomegalovirus (CMV) infection associated with hemophagocytic lymphohistiocytosis (HLH) in an immunocompetent host meeting all eight HLH 2004 diagnostic criteria. Cureus 2017;9(3):e1070. 28409071 10.7759/cureus.1070 PMC5376153

[8] Atim-Oluk M Cytomegalovirus associated haemophagocytic lymphohistiocytosis in the immunocompetent adult managed according to HLH-2004 diagnostic using clinical and serological means only. Eur J Microbiol Immunol (Bp) 2013;3(1):81–9. 24265923 10.1556/EuJMI.3.2013.1.12 PMC3832083

[9] Tsuda H Shirono K Successful treatment of virus-associated haemophagocytic syndrome in adults by cyclosporin A supported by granulocyte colony-stimulating factor. Br J Haematol 1996;93(3):572–5. 8652375 10.1046/j.1365-2141.1996.d01-1707.x

[10] Suzuki T Takaya S Kunimatsu J GATA2 mutation underlies hemophagocytic lymphohistiocytosis in an adult with primary cytomegalovirus infection. J Infect Chemother 2020;26(2):252–6. 31350183 10.1016/j.jiac.2019.07.002

